# Outcome of patients with COVID-19 supported by veno-venous extracorporeal membrane oxygenation with major bleeding: a single centre experience

**DOI:** 10.1186/s12871-025-03380-9

**Published:** 2025-10-02

**Authors:** Simon Dubler, Michael Kowarik, Bettina Budeus, Tomas Habanik, Denise Zwanziger, Annabell Skarabis, Florian Espeter, Thorsten Brenner, Frank Herbstreit

**Affiliations:** 1 Department of Anesthesiology and Intensive Care Medicine, University Hospital Essen, University Duisburg-Essen, D-45147 Essen, Germany; 2 Institute of Cell Biology (Cancer Research), University of Duisburg-Essen, D-45147 Essen, Germany; 3 Department of Anaesthesiology and Intensive Care Medicine, Klinik Floridsdorf, Vienna, A- 1210 Austria; 4 Department of Endocrinology, Diabetology und Metabolism, Clinical Chemistry, Division of Laboratory Research, University Hospital Essen, University Duisburg- Essen, Essen, Germany

**Keywords:** COVID-19, ARDS, ECMO, Bleeding, Thrombosis, Intracranial haemorrhage

## Abstract

**Background:**

Patients with severe COVID-19 often require veno-venous extracorporeal membrane oxygenation (VV-ECMO) due to acute respiratory distress syndrome (ARDS). Major bleeding complications are common and linked to worse outcomes, though specific risk factors in COVID-19 remain unclear.

**Methods:**

A retrospective analysis of 151 critically ill patients with COVID-19 on VV-ECMO (March 2020–December 2021) was conducted. The primary outcome was major bleeding (fatal bleeding, haemoglobin drop ≥ 20 g/L^−1^ (1.24 mmol/L^−1^), or symptomatic bleeding in critical organs). Secondary outcomes included 90-day mortality, kidney replacement therapy, and disease severity.

**Results:**

Major bleeding occurred in 73/151 patients (48.3%). Only a longer ECMO duration [OR 1.32 (95% CI 1.14–1.53; *p* < 0.001)] was identified as an independent risk factor. Kidney replacement therapy independently influenced 90-day mortality [OR 4.48 (95% CI 1.83–10.98;*p* = 0.001). However, major bleeding, intracranial haemorrhage, higher burden of co-morbidity and mean aPTT before major bleeding were not associated with an increased 90-day mortality risk.

**Conclusion:**

Major bleeding events, including intracranial haemorrhage, are common in patients with COVID-19 being supported by VV-ECMO. However, our data does not demonstrate a direct association between major bleeding and increased 90-day mortality.

**Supplementary Information:**

The online version contains supplementary material available at 10.1186/s12871-025-03380-9.

## Background

Severe acute respiratory syndrome, coronavirus disease 2 (SARS-CoV-2) emerged globally in late 2019 and early 2020. The ensuing pandemic led to hundreds of millions of cases and an estimated death toll of over 7 million people worldwide [1]. While most patients experienced only mild infections, a significant proportion required intensive care treatment [2]. In particular, patients with severe coronavirus disease 2019 (COVID-19) often needed veno-venous extracorporeal membrane oxygenation (VV-ECMO) due to developing severe acute respiratory distress syndrome (ARDS). The prognosis for these patients was poor, with mortality rates exceeding 60% [3]. As a response to the pandemic, numerous centres initiated ECMO programs to meet increasing patient demands [4]. According to expert recommendations, ECMO centres should manage at least 20 patients per year to ensure high-quality clinical care [5]. Notably, a large retrospective analysis revealed that centres performing more than 50 procedures annually achieved the highest survival rates for COVID-19 ECMO patients [6].

A major challenge in ECMO therapy is the procedure’s high incidence of bleeding and thrombotic complications. Patients with COVID-19 were particularly prone to coagulation disorders, leading to an increased risk of pulmonary artery emboli and bleeding compared to patients without COVID-19 [7, 8]. While the optimal anticoagulation strategy for ECMO therapy in patients with COVID-19 remains a topic of debate [8–10], studies have not demonstrated that a more aggressive anticoagulation approach effectively reduces thrombotic complications [11, 12]. On the contrary, an increased incidence of haemorrhagic events has been reported in patients with COVID-19 compared to controls [13]. More specifically, major bleeding complications were frequently observed in ECMO patients and were often linked to a worse prognosis [6]. Despite these findings, individual risk factors for bleeding in patients with COVID-19 remain unclear. To address this gap, we conducted a monocentric, retrospective study to identify patients at risk for major bleeding events and to assess whether these events were associated with poorer outcomes.

## Methods

This monocentric, retrospective study was performed at the ICU of the Department of Anaesthesiology and Intensive Care Medicine, University Hospital Essen (Essen, Germany).

Patients admitted to the ICU between March 2020 and December 2021 with a confirmed SARS-CoV-2 infection (COVID-19) and a need for VV-ECMO support were included in the study. For the detection of SARS-CoV-2, a polymerase chain reaction (PCR) was used. Sampling was performed by using nasopharyngeal swabs in patients with a tracheostomy or bronchioalveolar lavage in intubated patients. The primary endpoint in this special cohort of patients was the occurrence of major bleeding.

Schulman et al. [14] defined major bleeding as the following:

- Deadly bleeding and/or.
- Symptomatic bleeding in/around a critical organ (intracranial, intraspinal, intraocular, retroperitoneal, intraarticular, pericardial, or intramuscular with compartment syndrome) with/or.
- Bleedings that cause a decrease in haemoglobin of 20 g/L^−1^ (1.24 mmol/L^−1^) or more or bleedings that require transfusions of two or more units of packed red cells.

Our secondary endpoints were 90-day mortality after ICU admission, need for kidney replacement therapy, and severity of disease. Disease severity was quantified using the Charlson Comorbidity Index (CCI) and a Sequential Organ Failure Assessment without the Glasgow Coma Scale (non-GCS SOFA) upon each patient’s ICU admission and a mean Simplified Acute Physiology Score 2 (SAPS II) for every patient was assessed on day 1 after ICU admission.

### Hospital setting

The University Hospital Essen is a tertiary care medical facility. The Department of Anaesthesiology and Intensive Care Medicine, which is part of the West German Centre for Infectious Diseases, operates a 22-bed ICU. All patient rooms are single rooms with advanced isolation capabilities, including air locks and negative pressure rooms. Our department serves as a referral centre for ARDS and ECMO in Germany, with over 100 ECMO procedures conducted annually. Most ECMO patients in our ICU were transferred from other hospitals. Our department also offers a mobile ECMO team, enabling initiation of ECMO support at the referring hospital and providing a transfer of these patients with ECMO to our ICU via a ground or air ambulance service.

### Treatment protocols

Herbstreit et al. [15] has already published a standardized diagnostic and treatment protocol for ARDS patients with ECMO at our department.

#### Specific treatment of SARS-CoV2

Since the publication of the RECOVERY trial in 2021 [16], all patients at the studied institution received 6 mg dexamethasone intravenously (IV) or orally for a minimum of six days and a maximum of ten days. Activated thromboplastin time (aPPT) monitoring was used for all patients on unfractionated heparin therapy. The targeted aPPT was 40–50 s during ECMO support, measured every 6 h. Patients with heparin-induced thrombopenia (HIT) or allergies received argatroban as a continuous infusion.

### ARDS

ARDS was defined according to the Berlin definitions [17].

### Sequential organ failure assessment without Glasgow coma scale (non-GCS SOFA)

The SOFA score describes the extent of organ dysfunction and predicts mortality in ICU patients [18]. Since assessment and documentation of the GCS in ICU patients is frequently uncertain and/or incomplete, we defined a modified non-GCS SOFA score for our data analyses, excluding the item GCS. This non-GCS SOFA score has already been used in another publication of this working group [19].

### Statistics

The data were stored in Excel (Microsoft^®^, Redmond, WA, USA) and then analysed in R (R Core Team (2022) R Core Team, Version 4.5.0 and 4.5.1. R: A Language and Environment for Statistical Computing; R Foundation for Statistical Computing: Vienna, Austria, 2022. Available online: https://www.R-project.org/ (accessed on 02 Feb 2025).) using the packages survminer (Kassambara, A. et al. Kassambara, A.; Kosinski, M.; Biecek, P.; Fabian, S. survminer: Drawing Survival Curves Using ‘ggplot’, R Package Version 0.4.9; R Foundation for Statistical Computing: Vienna, Austria, 2021. Available online: https://CRAN.R-project.org/package=survminer (accessed on 02 Feb 2025).), R package version 0.4.9), and survival (Therneau, T. (2021) [Therneau, T. A Package for Survival Analysis in R, R Package Version 3.1–13; R Foundation for Statistical Computing: Vienna, Austria, 2021. Available online: https://cran.r-project.org/web/packages/survival/index.html (accessed on 02 Feb 2025.] R package version 3.2–13), for survival analysis, and gtsummary [Daniel, D.S.; Whiting, K.; Curry, M.; Lavery, J.A.; Larmarange, J. Reproducible summary tables with the gtsummary package. R J.2021, 13, 570–580. to display the data in tables. Continuous data are presented as the median and quartils. Categorical variables are displayed as absolute and relative frequencies. The Mann–Whitney U-test or the chi-square test was used to calculate potential differences between the groups. Cox proportional hazards regression model with adjustment for potential confounders was used to identify risk factors for mortality (hazard ratio (HR)). Two-sided *p* < 0.05 was considered statistically significant for all analyses. To measure the influence of ICH in patients with major bleeding events on mortality (90-day ICU), cox proportional hazards regression model was done twice, since those two items cannot be put together.

## Results

**Fig. 1:**
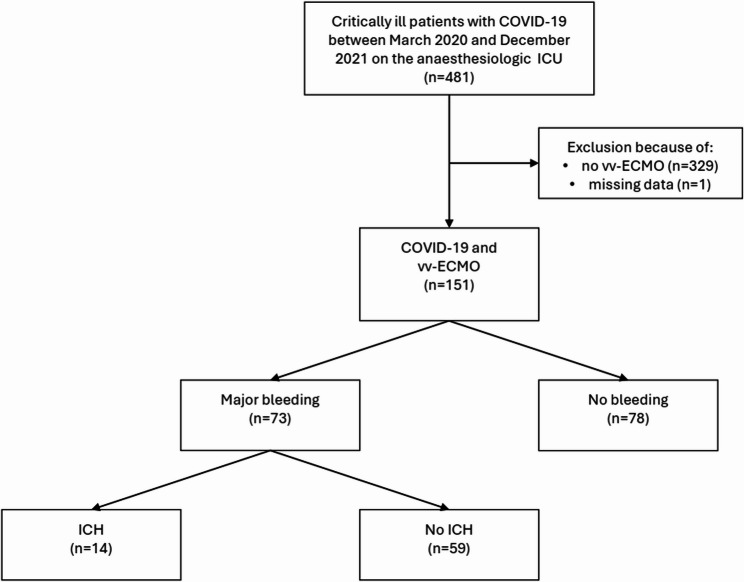
Flow chart of inclusion and exclusion criteria. Abbreviations: *COVID-19* (Corona-Virus-Disease-2019), *ICU* (Intensive Care Unit), *vv-ECMO* (veno-venous extracorporeal membrane oxygenation), *ICH* (intracranial haemorrhage)

Between March 2020 and December 2021, 481 patients with confirmed SARS-CoV-2 infection and COVID-19 were treated in our ICU. In total, 151/481 (31.4%) received VV-ECMO support. Hence, 151 patients were included in the final analysis. See Fig. 1 for the detailed study flow chart.

Of these patients, 73 (48.3%) suffered from major bleeding (MB) during VV-ECMO support, whereas the remaining 78 (51.7%) did not (the “non-bleeding” group). Of the MB events, 14 (19.2%) were intracranial haemorrhages (ICHs). The baseline characteristics of all included patients are displayed in Table 1. There was no difference in terms of age (*p* = 0.30), sex (*p* = 0.90) or mean aPTT before major bleeding events (*p* = 0.30) between patients with MB and the non-bleeding group. The length of ECMO support was longer in patients with MB than those without MB (480 vs. 262 h, respectively; *p* < 0.001). Additionally, patients with MB spent more time in ICU than patients without MB (21 vs. 15 days; *p* < 0.005). SAPS II Score on day 1 after ICU admission did not differ between the two groups (*p* = 0.90). There was, however, no difference in the patients’ 90-day mortality (MB (72%) vs. non-bleeding (68%); *p* = 0.06).

**Table 1:** Baseline characteristics of major bleeding and non-bleeding patients

	Major bleeding (*n* = 73)	Non-bleeding (*n* = 78)	*p*
Demographics			
Age [years]^#^	54 (49, 59)	55 (39, 61)	0.30
Male	50 (68%)	55 (71%)	0.90
Co-morbidity			
BMI [kg/m^2^]^#^	30 (28, 37)	32 (28, 36)	0.90
CCI^#^	1 (1, 2)	1.5 (1, 2)	0.90
SAPS II day 1^#^	33 (25, 41)	33 (25, 39)	0.90
aPTT (sec)*	45 (42, 48)	47 (44, 53)	0.09
Mean aPTT_before_Major Bleeding	47 (44, 53)	45 (42, 48)	0.30
HIT^#^	3 (4.1%)	4 (5.1%)	0.80
Horowitz Index^#^	114 (72, 182)	93 (71, 133)	0.15
nonGCS SOFA^#^	9 (7, 10)	8 (7, 10)	0.80
Length of ECMO support^#^ [hours]	480 (279, 673)	262 (161, 397)	< 0.001
Complications			
Bacteraemia	60 (82%)	56 (72%)	0.20
Candidemia	10 (14%)	9 (12%)	0.70
Kidney replacement therapy	51 (70%)	51 (66%)	0.60
Outcome			
Length of ICU stay [days] ^#^	21 (14, 34)	15 (11, 22)	< 0.005
Survivors Day 90	13 (18%)	25 (32%)	0.06

Data are presented as n (%)

*Abbreviations*: *BMI* (Body Mass Index), *CCI* (Charlson Co-morbidity Index), *SAPS II* (Simplified Acute Physiology Score II), *aPTT* (Activated partial thromboplastin time), *HIT* (Heparin induced thrombocytopenia), *Horowitz Index* (FiO_2_/paO_2_) during ICU admission, *SOFA* (Sequential Organ Failure Assessment), *ECMO* (Extracorporeal membrane oxygenation), *ICU* (Intensive Care Unit)

^#^Values are presented as median (IQR)

^*^Values are presented as mean

Looking at the 90-day survival (see Table 2), survivors were younger and more often female compared to non-survivors (49 years vs. 56 years, *p* < 0.001, and 47% vs. 25%, *p* < 0.05, respectively). Additionally, patients in the non-survivor group showed a higher burden of comorbidity, shown by the CCI score (2 vs. 1 points, *p* < 0.005). The severity of disease was also higher in the group of non-survivors, with higher SAPS II scores on day 1 after ICU admission (34 vs. 29 points, *p* < 0.05). Non-survivors also showed a significantly higher need for kidney replacement therapy. Mean aPTT before major bleeding did not differ between the two groups (44 vs. 46 s, *p* = 0.20) MB events differed between the two groups but were not statistically significant, with 53% of survivors and 34% of non-survivors experiencing MB (*p* = 0.06).

**Table 2:** Baseline characteristics of survivors and non-survivors 90 days after ICU admission

*n* = 151	Survivors (*n* = 38)	Non-survivors (*n* = 113)	*p*
Demographics			
Age [years]^#^	49 (35, 55)	56 (49, 62)	< 0.001
Male	20 (53%)	85 (75%)	< 0.05
Co-morbidity			
BMI [kg/m^2^]^#^	31 (28, 35)	31 (28, 37)	0.3
CCI^#^	1.00 (0.00, 2.00)	2.00 (1.00, 2.00)	< 0.005
SAPS II day 1^#^	29 (22, 36)	34 (28, 41)	< 0.05
aPTT (sec)*	44 (40, 47)	47 (44, 53)	0.05
Mean aPTT before_Major Bleeding	44 (41, 48)	46 (43, 53)	0.20
Horowitz Index^#^	112 (85, 164)	98 (69, 167)	> 0.9
nonGCS SOFA^#^	8 (7, 10)	9 (7, 10)	0.6
Length of ECMO support^#^ [hours]	283 (186, 491)	349 (180, 509)	0.6
Complications			
Bacteraemia	31 (82%)	85 (75%)	0.5
Candidemia	8 (22%)	11 (9.8%)	0.12
Kidney replacement therapy	15 (41%)	87 (77%)	< 0.001
Major bleeding	13 (34%)	60 (53%)	0.06
ICB	0 (0%)	14 (12%)	< 0.05

Data are presented as n (%)

*Abbreviations*: *BMI* (Body Mass Index), *CCI* (Charlson Co-morbidity Index), *SAPS II* (Simplified Acute Physiology Score II), *aPTT* (Activated partial thromboplastin time), *Horowitz Index* (FiO_2_/paO_2_) during ICU admission, *SOFA* (Sequential Organ Failure Assessment), *ECMO* (Extracorporeal membrane oxygenation), *ICB* (Intracerebral bleeding)

^#^Values are presented as median (IQR)

^*^Values are presented as mean

**Fig. 2:**
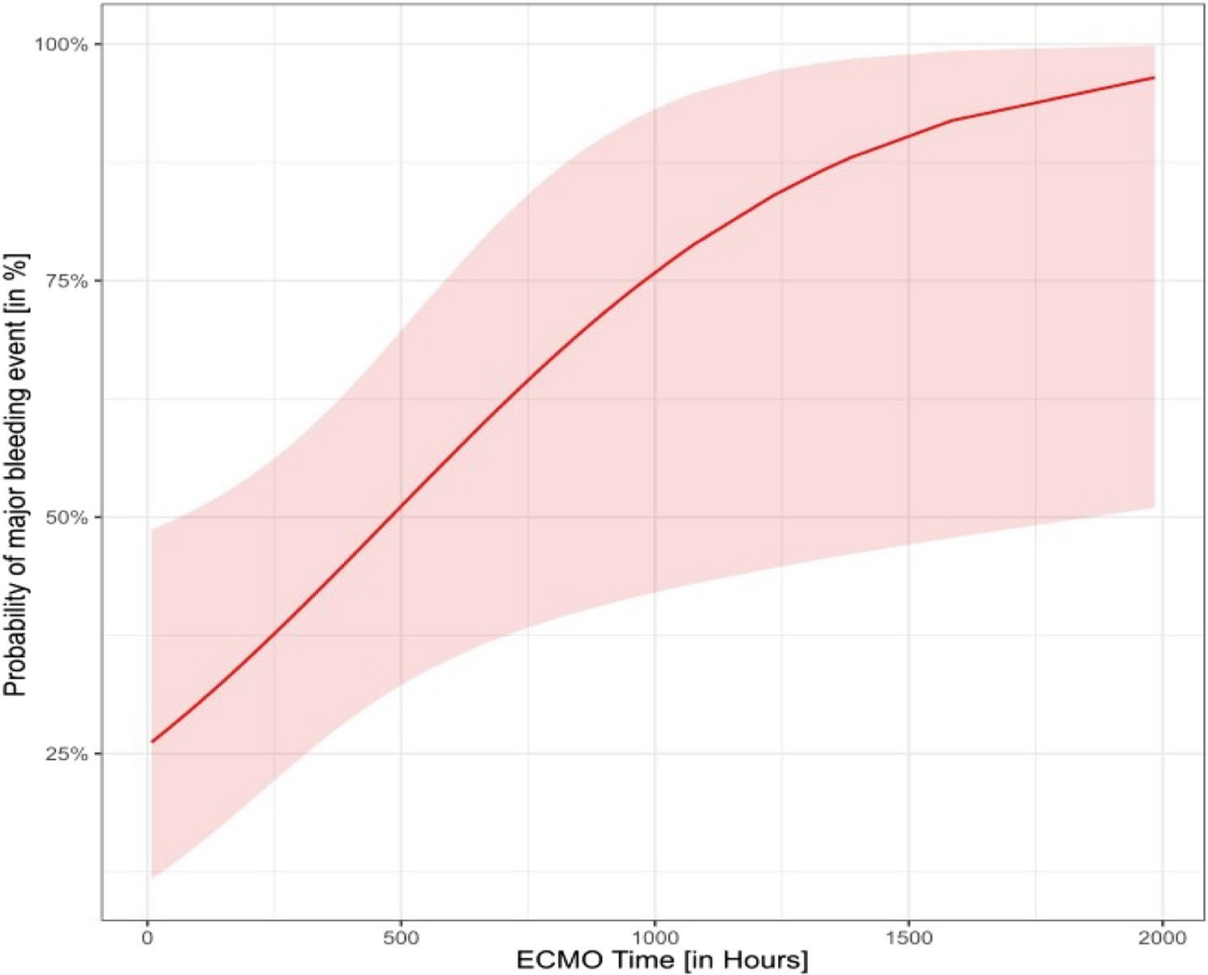
Probability of bleeding events in relation to ECMO duration. Abbreviations:*vv-ECMO* (veno-venous extracorporeal membrane oxygenation)

**Fig. 3:**
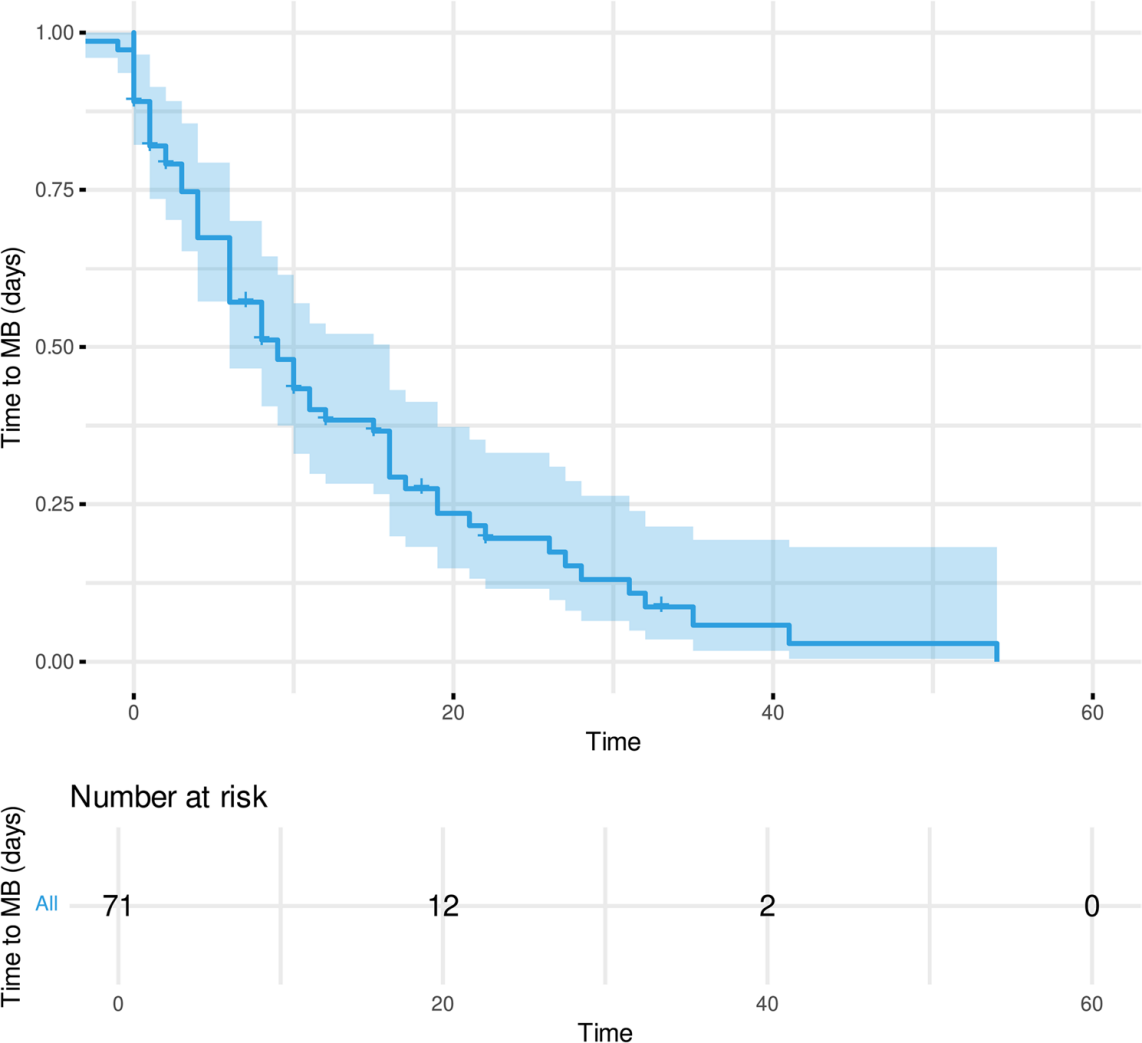
Kaplan-Meier curve: Time to first Major bleeding event relative to ECMO initiation. Abbreviations: *MB* (Major Bleeding).

To identify factors independently influencing the occurrence of MB, a univariable and multivariable regression analysis were performed (see Table 3). For the purposes of this study, a longer duration of ECMO support was the only independent risk factors for a MB event during an ICU stay, with ORs of 1.32 (95% CI 1.14–1.53; *p* < 0.001), whereas Mean aPTT before major bleeding was not (*p* = 0.39) Fig. 2 illustrates the relationship between ECMO duration and the risk of bleeding events. After 500 h of ECMO support, the risk of developing MB events exceeded a 50% probability. To estimate the time between the start of ECMO-support and the occurrence of first bleeding event, a Kaplan-Meier curve was created (Fig. 3).

**Table 3:** Independent risk factors for development of major bleeding events

*n* = 151	Odds Ratio (95% CI)	*p*
Univariable Analysis		
Age	1.01 (0.99, 1.04)	0.31
Sex	1.10 (0.55, 2.20)	0.79
SAPS II day 1	1.00 (0.98, 1.03)	0.88
Mean aPTT before Major Bleeding	1.03 (0.98, 1.07)	0.21
ECMO time	1.28 (1.12, 1.47)	< 0.001
Kidney replacement therapy	1.18 (0.59, 2.35)	0.63
**Multivariable Analysis** ^1^		
Age	1.03 (0.99, 1.06)	0.12
Sex	1.21 (0.56, 2.61)	0.63
SAPS II day 1	1.00 (0.97, 1.03)	0.88
Mean aPTT before Major Bleeding	0.98 (0.95, 1.02)	0.39
ECMO time	1.32 (1.14, 1.53)	< 0.001
Kidney replacement therapy	1.22 (0.56, 2.61)	0.62

*Abbreviations*: *SAPS II* (Simplified Acute Physiology Score II), *aPTT* (Activated partial thromboplastin time), *ECMO* (Extracorporeal membrane oxygenation)

^1^Colinearity values: Age (1.291890), Sex (1.051283), Kidney replacement therapy (1.076177), SAPS day 1 (1.297365), Mean aPTT before Major Bleeding (1.061526), ECMO duration (1.043612)

A univariate and multivariate analysis were performed to determine independent risk factors for the 90-day mortality in this special cohort of patients (see Table 4). Only the need for kidney replacement therapy autonomously influenced 90 day mortality in both analysis, with and without ICH. SAPS II on day 1, mean aPTT before major bleeding, CCI, MB and ICH did not affect the 90-day mortality rate in the multivariate analysis.

**Table 4:** Independent risk factors for death after 90 days on ICU

*n* = 151	Univariate Analysis		Multivariate Analysis (with major bleeding)		Multivariate Analysis (with ICH)	
	Odds Ratio (95% CI)	*p*	Odds Ratio (95% CI)	*p*	Odds Ratio (95% CI)	*p*
Age	1.05 (1.02, 1.08)	< 0.05	1.00 (0.95, 1.05)	0.93	1.00 (0.95, 1.05)	0.96
Sex	0.37 (0.17, 0.79)	< 0.05	0.73 (0.26, 2.01)	0.54	0.82 (0.30, 2.27)	0.71
SAPS II mean	1.21 (1.13, 1.30)	< 0.001	1.19 (1.08, 1.32)	< 0.001	1.13 (1.04, 1.23)	< 0.005
ECMO time	0.97 (0.86, 1.08)	0.53	1.02 (0.87, 1.19)	0.82	1.04 (0.91, 1.20)	0.56
Kidney replacement therapy	4.91 (2.23, 10.8)	< 0.001	2.18 (0.76, 6.22)	0.15	2.14 (0.73, 6.25)	0.16
Major Bleeding^a^	2.18 (1.01, 4.68)	< 0.05	1.42 (0.45, 4.46)	0.55	xx	xx
ICH^b^	Inf (0.00, Inf)	0.99	xx	xx	2.79 (0.00, ∞)	0.99

*Abbreviations*: *SAPS II* (Simplified Acute Physiology Score II), *ECMO* (Extracorporeal membrane oxygenation), *ICH* (Intracranial haemorrhage)

^a^with major bleeding

^b^with ICH

## Discussion

In our study, 151 patients with COVID-19 were treated with VV-ECMO. The 90-day survival in all included patients was 25%. Only a longer ECMO duration increased the risk for MB events. Notably, MB events or ICHs (one of the most dangerous MB events) did not negatively affect the 90-day mortality in this special population.

Despite this finding, bleeding among patients with ECMO support remains a common and dangerous complication. The prevalence of bleeding events varies between different studies. Data from an ELSO registry analysis revealed that up to 37% of patients with VV-ECMO suffer from at least one bleeding event during their ICU stay [20]. In another international observational study (the PROTECMO study), 52.5% of patients experienced at least one bleeding event [9]. The difference in the prevalences of bleeding events depends highly on the applied definition for this severe complication. In the ELSO registry analysis, bleeding was divided into surgical bleeding (cardiac tamponade, cannulation, and surgical site bleeding) and medical bleeding (pulmonary, gastrointestinal, or ICH). In the PROTECMO study, bleeding episodes were assessed using a set of predefined sites (cannulation, cerebral, gastric, ear and/or nose and/or throat, intestinal, intraabdominal, intrathoracic, tracheal and/or pulmonary, and urinary tract), whereas severity was standardized using four categories according to a modified version of the Bleeding Academy Research Consortium score [21]. In the original ECMO for ARDS publication by Alain Combes in 2018 [22], bleeding events leading to a transfusion of red blood cells occurred in 46% of patients.

As SARS-CoV-2 infections became a pandemic, a substantial percentage of patients with COVID-19 needed intensive care treatment, depending on their country and region [23]. Due to the different pulmonary pathobiology and vascular angiogenesis of COVID-19 [24], intensivists started to increase their anticoagulation targets [8]. This COVID-19–associated microangiopathy led to significantly higher rates of thrombotic events compared to other viral infections and thus possibly higher anticoagulation targets set by ECMO providers [7]. Unfortunately, this also led to higher rates of ICHs [25]. In an observational study of 309 patients with COVID-19 with VV-ECMO in the United Kingdom [26], 93 (30.1%) patients experienced MB, of whom 30 (32.3%) developed ICH. In this study, MB was an independent risk factor for mortality, with a hazard ratio (HR) of 3.9 (95% CI 2.6–5.8). Another study retrospectively analysed 1,248 patients with COVID-19 who underwent VV-ECMO worldwide. In this study, 24% of patients showed bleeding complications, and 19% patients suffered from ICHs. Again, major bleeding was associated with higher ICU mortality (HR 1.6, 95% CI 1.28–1.99) [27]. Additionally, a nationwide study from France showed 29% of patients with COVID-19 who underwent VV- and veno-arterial-ECMO experienced bleeding events. In the multivariate analysis, bleeding was independently associated with mortality [28].

None of the mentioned multicentre studies stated the experience or the level of the involved centres when it comes to ECMO support. as the CESAR trial showed before the COVID-19 pandemic [29] that certain ECMO centres, particularly ones with experienced staff, lead to better patient outcomes. When it comes to critically ill patients with COVID-19, Herrmann et al. [6] clearly showed the relationship between ECMO experience and outcomes, finding higher odds of ICU survival in high-volume ECMO centres. In this German multicentre study among patients with COVID-19, patients in high-volume ECMO centres (> 50 ECMO/year) presented with less bleeding or thrombotic events (45.2%) compared to intermediate- (20–40 ECMO/year) and low-volume (< 20 ECMO/year) centres (77.2% and 72.9% respectively, *p* < 0.001). This difference is also reflected in mortality: High-volume ECMO centres revealed the highest survival rate compared to intermediate- or low-volume ECMO centres (38.3% vs. 29.5% vs. 19.8%, *p* = 0.0024, respectively).

Nonetheless, the 90-day survival rate of all patients included in our cohort was as low as 25% (38/151). One reason for this result might lie in the time of patient allocation. Our study looked at patients almost exclusively during the second wave of the COVID-19 pandemic. The survival rates during the second wave have already been shown to have been significantly lower compared to the first wave. Our own institution showed survival rates of 65.6% in patients with COVID-19 (with and without VV-ECMO) during the first wave [15]. Herrmann and colleagues [6] showed in their retrospective analysis among multiple German ECMO centres that the survival rate during the first wave of the pandemic (40.3%) decreased to 27.9% (*p* = 0.0019) during the second wave [6]. Similar results were shown by Jordi Riera and the ECMOVIBER study group [3], whose research revealed that survival rates dropped from 58.9% (first wave) to 39.9% (second wave), *p* = 0.001. In a research letter, published in critical care in 2021 by Christian Karagiannidis and colleagues [30], who looked on the mortality throughout the first three waves of the pandemic, they found nearly the same high mortality among 3.397 patients in 213 hospitals (68% mortality) as in our study. Interestingly, they found that non-survivors were significantly older (53 years) compared to survivors (59 years). The same is true for our cohort, where survivors were significantly younger than non-survivors (49 years vs. 56 years, *p* < 0.001). In the original ELSO report published by Barbaro et al. in 2021 [4], mortality rates were much less but the patients were also much younger (51 years in the median). Compared to other countries in the world, Germany has put older and sicker patients on ECMO therapy, probably because the resources and ECMO-machines were available. Another potential factor could be the burden of comorbidities in our cohort. 77% of the non-survivors needed kidney replacement therapy and showed a SAPS II score on day 2 as high as 34. The use of renal replacement therapy was also associated with a higher chance of non-survival in the study by Herrmann et al. [6], whereas the direct link between ECMO and acute kidney injury is still unknown.

Another important finding was that, in our cohort, MB was not a risk factor for 90-day mortality. As intimated above, this result might mirror the high experience in our institution, which has had more than 100 ECMO therapies/year over the last decade. Whebell et al. [31] supports this thesis with their recent analysis of 243 COVID-19 ECMO patients in the United Kingdom. They found that referring patients to a specialized ECMO centre reduced their absolute mortality from 44% to 25.8% (*p* < 0.001). In a multicentre study from Paris, Lebreton and colleagues [32] also showed patients with COVID-19 treated in in centres managing at least 30 VV-ECMO procedures per year improved their 90-day mortality significantly (OR 2.98, CI 95% 1.46–6.04).

Survivors were significantly younger than non-survivors in our cohort (49 vs. 56 years, *p* < 0.001). This finding is in line with the literature. Older patients already demonstrated worse general outcomes long before the SARS-CoV-2 pandemic [33, 34], as age is one factor to consider in the two popular scores predicting mortality in ECMO patients: the PRESERVE score [35] and the RESP [36] score. Higher age, renal impairment, and an overall higher severity of disease as independent risk factors for mortality, as presented in our study, were also seen by other investigators studying critically ill patients with COVID-19 undergoing VV-ECMO [6, 26, 32, 37]. Because of these myriad influencing factors, decision making for or against ECMO support should incorporate numerous variables. Every patient must be treated individually, and the risk versus benefits, including those related to available resources, must be carefully weighted up against each other.

In our cohort of patients, a longer duration of ECMO independently increased the risk of bleeding by 1.32 (OR 1.31 (CI 95% 1.14–1.53, *p* < 0.001)). These results are comparable to those presented in the current literature, where longer ECMO support has been identified as an independent risk factor for bleeding [20, 28, 38]. Interestingly, patients in the already cited nationwide registry study from France showed a shorter duration of ECMO support, with a median of 11 days for all included patients and 12 days and 13 days for patients with bleeding and no bleeding events, respectively [28]. Our cohort of patients received ECMO support for 20 days in the group with MB and 11 days in the group without any bleeding events, a much longer duration than those noted in other studies. The ELSO registry analysis [20] also revealed shorter ECMO support, with a mean duration of 8 days for all included patients. Overall, then, question remains if the influence of the ECMO duration is directly connected to the ECMO machine itself or a longer duration of ECMO only reflects the severity of illness of the patients involved.

ICH is one of the worst bleeding complications during ECMO support. Before the COVID-19 pandemic, ICH seemed to be an infrequent complication. In an ELSO registry study published in 2017 [39], only 3.6% of patients with VV-ECMO suffered from ICH, and these patients showed a high mortality rate of 76%. ICH accounted for 14 out of 73 (19.2%) MB events in our cohort, with 12 of these patients (85.7%) dying before day 31 after ICU admission and 100% dying before day 50. These results are comparable to those identified other COVID-19 research. A multicentre, retrospective analysis by Seeliger et al. [25] comparing the occurrence of ICH in ECMO patients with COVID-19 and other viral infections revealed a 20% occurrence of ICH in the COVID-19 group versus 6% in the control group. Notably, 88% of all patients with an ICH did not survive their ICU stay. A systematic review and meta-analysis published in 2023 by Lannon et al. [13] that included 4,000 ECMO patients found an increased mortality rate among patients with COVID-19 with an ICH (64%) compared to patients without an ICH (41%).

The direct impact of higher anticoagulation levels and bleeding events in patients with ECMO was already shown in the PROTECMO trial [9] during the SARS-CoV-2 pandemic. In a multiple penalized Cox proportional hazard model, higher aPTT was a potentially modifiable risk factor for the first episode of bleeding (for 20-seconds increase; hazard ratio, 1.07). The authors stated that reducing anticoagulation targets (without mentioning the new targets) may be an effective way to minimize the risk of bleeding in ECMO-patients. Our centre used a little lower aPTT-target (40–50 s) as used in the PROTECMO trial (40–60 s). This may explain why the mean aPTT before major bleeding was not an independent risk factor for major bleeding events or mortality in our study although non-survivors showed a slightly higher aPTT (47 s) which was still within the targeted range (40–50 s). Mansour et al. [28] provided data that MB complications in COVID-19 ECMO patients are associated with a worse outcome. Most of the patients included in the study were managed with therapeutic anticoagulation. This is alarming since there is profound evidence from large randomized controlled trials that therapeutic anticoagulation is not beneficial for patients with COVID-19 [12]. Relatedly, Hofmaenner et al. [40] showed that a less aggressive anticoagulation strategy in these patients might be beneficial. Their study analysed 141 patients with COVID-19 who underwent ECMO support and found that patients with lower anticoagulation targets (lower anti-Xa activity) showed a strikingly lower incidence of ICH (8%) versus patients with higher anti-Xa activity levels (34%).

This study has certain limitations. First, bleeding events were diagnosed when they became symptomatic or were discovered with imaging techniques. Thus, the exact time of bleeding cannot be pinpointed in most cases. Second, due to the retrospective design, the cohort of patients is probably too small to adequately assess the risk factors related to major bleeding in this cohort. The study suffers from potential biases with some patients dying from other courses before a potential bleeding event could have occurred. This is a weakness of such trials. Other patients recovered and were weaned from ECMO support before suffering a potential bleeding event.

To form a deeper and more well-rounded knowledge base on this topic, future research should not only focus on anticoagulation strategies but also on patient-centred ECMO care. Anticoagulation is one thing, but managing its complications is another. Life-threatening bleeding complications such as ICH or massive haemorrhages need to be recognized and responded to by a trained staff with an approach that involves both nurses and doctors. Nevertheless, even in highly experienced centres, the development of ICH consistently leads to patient mortality, as shown in our own institution. On the other hand, other severe bleeding complications can be handled if a whole team comprised of critical care specialists, surgeons, and the blood bank get involved. Through such an approach, the outcomes of this very special cohort of critically ill patients can be improved [41].

## Supplementary Information

Supplementary Material 1

## Data Availability

No datasets were generated or analysed during the current study.

## Abbreviations

SARS: CoV–2–Severe acute respiratory syndrome coronavirus 2
COVID: 19–Coronavirus disease 2019
VV: ECMO–Veno–venous extracorporeal membrane oxygenation
ARDS: Acute respiratory distress syndrome
ICU: Intensive care unit
ECMO: Extracorporeal membrane oxygenation
CCI: Charlson Comorbidity Index
SOFA: Sequential Organ Failure Assessment
non: GCS SOFA–Sequential Organ Failure Assessment without the Glasgow Coma Scale
SAPS II: Simplified Acute Physiology Score 2
aPPT: Activated partial thromboplastin time
IV: Intravenous
HIT: Heparin–induced thrombopenia
MB: Major bleeding
ICH: Intracranial haemorrhage
ELSO: Extracorporeal Life Support Organization
HR: Hazard ratio
OR: Odds ratio
CESAR: Conventional ventilatory support versus extracorporeal membrane oxygenation for severe adult respiratory failure
PRESERVE: Predicting Death for Severe ARDS on VV–ECMO
RESP: Respiratory ECMO Survival Prediction
PROTECMO: Protective ventilation during extracorporeal membrane oxygenation
