# Supplementary material for: Outcome of patients with COVID-19 supported by veno-venous extracorporeal membrane oxygenation with major bleeding: a single centre experience
Source: BMC Anesthesiol. 2025 Oct 2;25:477. doi: 10.1186/s12871-025-03380-9 (PMC12490068; doi:10.1186/s12871-025-03380-9)
Supplement: Supplementary file 1 — Supplementary Material 1 [file 12871_2025_3380_MOESM1_ESM.pdf]

Table 4: Independent risk factors for death after 90 days on ICU

n = 151	Univariate Analysis		Multivariate Analysis (with major bleeding[a,1])		Multivariate Analysis (with ICH[b,2])	
	Odds Ratio (95% CI)	*p*	Odds Ratio (95% CI)	*p*	Odds Ratio (95% CI)	*p*
Age	1.05 (1.02, 1.08)	0.001	1.03 (0.98, 1.08)	0.20	1.04 (0.99, 1.09)	0.09
Sex	0.37 (0.17, 0.79)	0.01	0.43 (0.17, 1.04)	0.06	0.52 (0.21, 1.30)	0.16
SAPS II day 1	1.03 (1.00,1.06)	<0.05	1.00 (0.95, 1.04)	0.87	0.99 (0.95, 1.07)	0.80
Mean aPTT before Major Bleeding	1.03 (0.98, 1.07)	0.21	1.01 (0.96, 1.07)	0.65	0.98 (0.95, 1.02)	0.39
ECMO time	0.97 (0.86, 1.08)	0.97	0.96 (0.84, 1.12)	0.64	1.02 (0.90, 1.17)	0.73
Kidney replacement therapy	4.91 (2.23, 10.80)	<0.001	4.48 (1.83, 10.98)	0.001	5.15 (2.05, 12.98)	<0.001
CCI	1.54 (1.10, 2.16)	0.12	1.34 (0.87, 2.07)	0.19	1.31 (0.86, 1.99)	0.21
Major Bleeding[a]	2.18 (1.01, 4.68)	<0.05	2.13 (0.75, 6.09)	0.16	xx	xx
ICH[b]	32.51 (0.25, 4303.08)	0.16	xx	xx	<0.001 (0.00, Inf)	0.99

Abbreviations: *SAPS II* (Simplified Acute Physiology Score II), *aPTT* (activated partial thromboplastin time), *ECMO* (Extracorporeal membrane oxygenation), CCI (Charlson-Comorbidity-Index), *ICH* (Intracranial haemorrhage)

[1]collinearity values: Age (1.844417), Sex (1.044510), Kidney replacement therapy (1.100463), SAPS II day 1 (1.437158), Mean aPTT befor Major Bleeding (1.079698), ECMO time (1.211447), Major bleeding (1.485533), CCI (1.539770)

[2]collinearity values: Age (1.655534), Sex (1.040924), Kidney replacement therapy (1.119468), SAPS II day 1 (1.306521), Mean aPTT before Major Bleeding (1.087834), ECMO time (1.079384), ICB (1.000000)
